# Genetic Variation in *ABCB1*, *ADRB1*, *CYP3A4*, *CYP3A5*, *NEDD4L* and *NR3C2* Confers Differential Susceptibility to Resistant Hypertension among South Africans

**DOI:** 10.3390/jpm14070664

**Published:** 2024-06-21

**Authors:** Jonathan N. Katsukunya, Erika Jones, Nyarai D. Soko, Dirk Blom, Phumla Sinxadi, Brian Rayner, Collet Dandara

**Affiliations:** 1 Division of Human Genetics, Department of Pathology, Institute of Infectious Disease and Molecular Medicine (IDM), Faculty of Health Sciences, University of Cape Town, Cape Town 7700, South Africa; ktsjon001@myuct.ac.za (J.N.K.); nyarai.soko@uct.ac.za (N.D.S.); 2 SAMRC/UCT Platform for Pharmacogenomics Research and Translation, South African Medical Research Council, Cape Town 7501, South Africa; erika.jones@uct.ac.za (E.J.); dirk.blom@uct.ac.za (D.B.); phumla.sinxadi@uct.ac.za (P.S.); brian.rayner@uct.ac.za (B.R.); 3 Department of Medicine, Division of Nephrology and Hypertension, Groote Schuur Hospital and Faculty of Health Sciences, University of Cape Town, Cape Town 7700, South Africa; 4 Department of Pharmaceutical Technology, School of Allied Health Sciences, Harare Institute of Technology, Harare P.O. Box BE 277, Zimbabwe; 5 Department of Medicine, Division of Lipidology and Cape Heart Institute, Groote Schuur Hospital and Faculty of Health Sciences, University of Cape Town, Cape Town 7700, South Africa; 6 Department of Medicine, Division of Clinical Pharmacology, Groote Schuur Hospital and Faculty of Health Sciences, University of Cape Town, Cape Town 7700, South Africa

**Keywords:** pharmacogenomics, hypertension, resistant hypertension, Africans, antihypertensive drugs

## Abstract

Resistant hypertension (RHTN) prevalence ranges from 4 to 19% in Africa. There is a paucity of data on the role of genetic variation on RHTN among Africans. We set out to investigate the role of polymorphisms in *ABCB1*, *ADRB1*, *CYP3A4*, *CYP3A5*, *NEDD4L,* and *NR3C2*, on RHTN susceptibility among South Africans. Using a retrospective matched case–control study, 190 RHTN patients (cases: blood pressure (BP) ≥ 140/90 mmHg on ≥3 anti-hypertensives or BP < 140/90 mmHg on >3 anti-hypertensives) and 189 non-RHTN patients (controls: <3 anti-hypertensives, BP < 140/90 or ≥140/90 mmHg), 12 single nucleotide polymorphisms were genotyped using polymerase chain reaction–restriction fragment length polymorphism (PCR-RFLP), quantitative PCR and Sanger sequencing. Genetic association analyses were conducted using the additive model and multivariable logistic regression. Homozygosity for *CYP3A5* rs776746C/C genotype (*p* = 0.02; OR: 0.44; CI: 0.22–0.89) was associated with reduced risk for RHTN. Homozygous *ADRB1* rs1801252G/G (*p* = 0.02; OR: 3.30; CI: 1.17–10.03) and *NEDD4L* rs4149601A/A genotypes (*p* = 0.001; OR: 3.82; CI: 1.67–9.07) were associated with increased risk for RHTN. Carriers of the of *ADRB1* rs1801252—rs1801253 G–C haplotype had 2.83-fold odds of presenting with RHTN (*p* = 0.04; OR: 2.83; CI: 1.05–8.20). These variants that are associated with RHTN may have clinical utility in the selection of antihypertensive drugs in our population.

## 1. Introduction

More than 1 billion people globally are living with hypertension [1] and between 10 and 20% progress to resistant hypertension (RHTN) [2]. RHTN affects between 4 and 19% and 13% of people with hypertension in Africa and South Africa, respectively [3,4]. RHTN is characterized by poor response to antihypertensive medication and is a relevant outcome of interest in pharmacogenomic research. RHTN is defined as either a blood pressure (BP) that remains above target (≥140/90 mmHg) while taking more than three antihypertensive drugs including a diuretic or a controlled BP (<140/90 mmHg) while taking four or more antihypertensive drugs in adherent patients [5].

Genetic factors have been implicated in the development of RHTN [6], accounting for up to 50% [7] of poor BP treatment responses [8,9]. Genetic variation may play a significant role in the way patients respond to six classes of commonly prescribed antihypertensive drugs: calcium channel blockers (CCBs), diuretics, angiotensin-converting enzyme inhibitors, angiotensin receptor blockers, mineralocorticoid receptor antagonists, and β-blockers [10,11].

In South Africa, hydrochlorothiazide (HCTZ), amlodipine, enalapril, losartan, atenolol, and spironolactone are examples of commonly used antihypertensive drugs [12]. Studies have shown that single nucleotide polymorphisms (SNPs) in *ABCB1*, *ADRB1*, *CYP3A4*, *CYP3A5*, *NEDD4L,* and *NR3C2* genes influence treatment outcomes. ABCB1 (or P-glycoprotein) is involved in the clearance of amlodipine and SNPs in the *ABCB1* gene, c.3435T>C (rs1045642, p.Ile1145=) and c.2677C>A (rs2032582, p.Ser893Ala) have been shown to influence clearance rates of amlodipine in Asian populations [13]. CYP3A4 and CYP3A5 enzymes are also involved in the clearance of amlodipine, and SNPs have been found to affect both the pharmacokinetics of amlodipine and BP response in Asian and African American populations [9,13]. Among Europeans, for the *CYP3A5* c.219-237T>C (rs774756 or *3) SNP, the T allele carriers were found to have an increased likelihood of switching from dihydropyridine CCBs to other antihypertensive medications [14]. ADRB1 (or β1-adrenergic receptor) is the target site for β-blockers. SNPs have been reported to affect the sensitivity of the receptors to β-blockers thereby influencing their efficacy. For example, Guerra et al. (2022), reported on the association of the *ADRB1* c.145A>G (rs1801252, p.Ser49Gly) and c.1165G>C (rs1801253, p.Gly389Arg) polymorphisms with differential response in heart failure patients taking β-blockers [15]. NEDD4L is the principal regulator of the activity of sodium ion channels in the kidney [16] and SNPs have been shown to affect response to HCTZ. For example, *NEDD4L* c.49-16229G>A (rs4149601) and c.-300G>C (rs292449) SNPs were associated with BP response or adverse cardiovascular outcomes in European patients on treatment with HCTZ [17,18]. The mineralocorticoid receptors (or NR3C2) are target sites for spironolactone and SNPs have been reported to directly affect spironolactone response and enalapril response indirectly. For example, *NR3C2* c.-2-358C>G (rs2070950) and c.538G>A (rs5522, p.Val180Ile) have been found to be associated with BP and heart failure responses in European [19] and Egyptian patients [20].

Little is known about the impact of these polymorphisms on Africans. Although important strides have been made in hypertension pharmacogenomics, such as the development of the International Consortium for Antihypertensive Pharmacogenomics Studies (ICAPS), findings from pharmacogenomic studies of hypertension still generate conflicting results across populations. In addition, according to the Pharmacogenomics Knowledgebase (PharmGKB^®^, Stanford, CA, USA) [13] and ICAPS, there is a lack of data on which genetic variants influence how African patients respond to antihypertensive treatment. Consequently, no antihypertensives have been annotated in the Clinical Pharmacogenetics Implementation Consortium (CPIC^®^, Stanford, CA, USA) pharmacogenetic guidelines or incorporated into dosing algorithms. Therefore, this highlights the need for harmonization across populations and extensive genomic characterization of African populations. Aggressive forms of hypertension, such as RHTN, are more common in people of African origin (i.e., Black African or Mixed Ancestry) [21], and pharmacogenes may play important roles in its development. In this study, we set out to investigate the role of genetic variation in six pharmacogenes, *ABCB1*, *ADRB1*, *CYP3A4*, *CYP3A5*, *NEDD4L*, and *NR3C2* in RHTN in South African patients.

## 2. Materials and Methods

### 2.1. Ethics Statement and Study Approval

This study is a sub-study to a larger project titled, “Pharmacogenomics of Cardiovascular Diseases: Focusing on Dyslipidemia and Hypertension (PRECODE)”, whose protocol was approved by the University of Cape Town Human Research Ethics Committee (UCT-HREC) with ethics clearance, “HREC 694/2020”, and was granted permission to recruit consenting participants for the study from the Hypertension Clinic, Groote Schuur Hospital, Cape Town, South Africa. This sub-study also has its own ethical approval which was granted by the UCT-HREC with ethics clearance, “HREC 141/2022”. The rights to privacy were respected and all medical information of the participants was kept confidential during the study.

### 2.2. Study Design and Setting

This was a retrospective matched case–control study. Patients attending the Hypertension Clinic at Groote Schuur Hospital, Cape Town, South Africa, from January 2006 to December 2016 and August 2021 to July 2022 who consented to DNA studies and access to their medical records were recruited into the study. Patients were matched 1:1 according to age, sex, and ethnicity utilizing a case–control design. All patients had hypertension and cases or controls were differentiated based on either having resistant hypertension (RHTN) or no RHTN. Cases were patients having RHTN, defined as (i) BP ≥ 140/90 mmHg on 3 or more antihypertensive drugs including a diuretic in optimal doses, or (ii) BP < 140/90 mmHg on 4 or more antihypertensive drugs. Adherence to antihypertensive therapy was confirmed by therapeutic drug monitoring (TDM) of amlodipine levels where possible (i.e., plasma amlodipine levels > 7.0 ng/mL) [22]. Controls were patients without RHTN, defined as BP < 140/90 mmHg or BP ≥ 140/90 mmHg on less than 3 antihypertensive drugs. The study included participants (i) with a confirmed diagnosis of primary or essential hypertension, (ii) of Black African and Mixed Ancestry (i.e., admixed population of South Africans resulting from intermarriages between Black African, European, San or Asian populations) descent, (iii) on at least one antihypertensive drug for at least a year prior, and (iv) >18 years at the time of recruitment. The study excluded participants (i) with a confirmed diagnosis of secondary hypertension, (ii) who were pregnant at the time of recruitment, (iii) with confirmed white coat hypertension or non-adherence, and (iv) not on treatment with antihypertensive drugs.

### 2.3. Sample Size Calculation

The study sample size was determined according to Naing et al. (2006) [23], which assumed the prevalence of RHTN to be 12.6% [4] and an alpha level of 0.05. According to the calculation, a sample size of at least 163 patients with RHTN would be required to provide at least 80% power. However, due to the study’s case–control nature, 163 more patients without RHTN would be required to serve as controls. This results in a total sample size of 326 patients. The Hypertension Clinic at Groote Schuur Hospital, South Africa, serves patients referred for specialist hypertension care and most have RHTN. Therefore, since there is no recent study estimating the true prevalence of RHTN in our setting, we acknowledge that a prevalence of 12.6% used in our calculation may be lower. As such, to account for this, our analysis included 379 patients (190 cases and 189 controls).

### 2.4. Genetic Characterization

Genomic DNA was extracted from 2 mL of venous blood from consenting participants using the Chemagic 360^®^ automated nucleic acid system (Chemagen Technologies, GMBH/Perkin Elmer Incorporated, Shelton, CT, USA) according to the manufacturer’s protocol. The quality of the extracted genomic DNA was assessed by electrophoresis using a 1% (*w*/*v*) agarose gel and the quantity of DNA was determined using spectrophotometry on a NanoDrop C1000 UV spectrophotometer (Thermofischer Scientific Corporation, Waltham, MA, USA). Genomic DNA of adequate quality and quantity was used for genotyping the 12 SNPs in six genes using polymerase chain reaction–restriction fragment length polymorphism (PCR-RFLP), TaqMan^®^ allelic discrimination assays, and Sanger sequencing.

Forward and reverse primers flanking regions of interest containing the SNPs were either designed or obtained from previous studies (Table S1). Each PCR was carried out in a 25 µL reaction volume containing 100 ng genomic DNA; 1X Green GoTaq Flexi^®^ Reaction Buffer (Promega Corporation, Madison, WI, USA); 0.4 mM of deoxynucleotide triphosphates (dNTPs) (Promega Corporation, Madison, WI, USA); 1.5 mM magnesium chloride (Promega Corporation, Madison, WI, USA); 0.2 mM of forward and reverse primers each (Inqaba Biotechnical industries (Pty) Ltd., Pretoria, South Africa), 1 U of GoTaq Flexi^®^ DNA Polymerase (Promega Corporation, Madison, WI, USA) and the volume made up to 25 uL with nuclease-free water. All PCRs were carried out using a SimpliAmp^™^ Thermal Cycler (Applied Biosystems, ThermoFisher Scientific, Life Technologies, Singapore). The reaction conditions were initial denaturation at 94 °C for 3 min, followed by 35 cycles of further denaturation at 94 °C for 30 s, annealing at temperatures specific for each SNP for 30 s (Table S1), initial extension at 72 °C for 30 s, and final extension at 72 °C for 10 min.

PCR products for *ABCB1* rs1045642, rs2032582, *ADRB1* rs1801252, rs1801253, *CYP3A5* rs776746, rs10264272, and rs41303343 SNPs were digested using *MboI*, *BSeYI*, *Sau96I*, *BstNI*, *SspI* and *DdeI* (for both rs10264272 and rs41303343) restriction enzymes, respectively (New England BioLabs^®^, Ipswich, UK). Each digest reaction was carried out in a 30 µL reaction volume containing, 10 µL of PCR product, 1X CutSmart™ Buffer (New England BioLabs^®^, Ipswich, UK), 3U restriction enzyme specific for each SNP, and nuclease-free water. All restriction enzyme digest reactions were incubated and inactivated on a SimpliAmp^™^ Thermal Cycler (Applied Biosystems, ThermoFisher Scientific, Life Technologies, Singapore) at temperatures and periods specific for each enzyme according to the manufacturer’s protocol (https://nebcloner.neb.com/#!/redigest (accessed on 16 May 2024)). Restriction enzyme digests were resolved using electrophoresis on a 3% (*w*/*v*) agarose gel stained with SafeView^TM^ Classic (Applied Biological Materials Inc., Richmond, BC, Canada) nucleic acid stain. Visualization was conducted on a UV transilluminator (UVITEC, Avebury House, Cambridge, UK) using the Fire Reader^®^ software for Windows (D-56-26.MX, France).

Validated TaqMan^®^ allelic discrimination genotyping assay kits, designed and synthesized by ThermoFisher Scientific Corporation (Waltham, MA, USA), were used for genotyping the *CYP3A4* rs2740574 (C___1837671_50), *NR3C2* rs5522 (C__12007869_20), rs2070950 (C___1594391_1_), *NEDD4L* rs4149601 (C___1424558_10) and rs292449 (C___1132327_10) SNPs. The reactions were set up in a total reaction volume of 10 µL containing 5 µL of 2X TaqPath^™^ ProAmp^™^ Master Mix (ThermoFisher Scientific Corporation Waltham, MA, USA), 0.5 µL of 20X TaqMan^®^ genotyping assay specific for each SNP and 45 ng of genomic DNA. All qPCR reactions were carried out on a CFX96 Touch Real-Time PCR Thermal Cycler (Bio-Rad Laboratories, Inc., CA, USA) and the reaction conditions were initial denaturation at 95 °C for 5 min followed by 50 cycles of further denaturation and annealing at 95 °C for 15 s and 60 °C for 1 min, respectively, then final elongation at 60 °C for 30 s.

Sanger sequencing was used to validate genotyping for the 12 SNPs. For each SNP, 10% of samples were selected making sure that all the representative genotypes were included. This approach ensured that the primary genotyping methods could accurately resolve each genotype group and provide robust validation across different genotypes without the need to sequence all the samples. Post-PCR clean-up using exonuclease I (ExoI) and thermosensitive alkaline phosphatase (FastAP^™^) enzymes from ThermoFisher Scientific (Waltham, MA, USA) was completed as per manufacturer’s protocol prior to sequencing. Each sequencing reaction was set up in a reaction volume of 10 µL, containing PCR product, BigDye™ Terminator v3.1 sequencing buffer, BigDye™ Terminator v3.1 Ready Reaction Mix (ThermoFisher Scientific, Waltham, MA, USA), and sequencing primer (Table S1). Sequencing reactions were carried out on a SimpliAmp™ Thermal Cycler (Applied Biosystems, Singapore) and the reaction conditions were initial denaturation at 96 °C for 1 min followed by 25 cycles of further denaturation at 96 °C for 10 s, annealing at 50 °C for 5 s and elongation at 60 °C for 4 min. Post-sequencing clean-up was performed according to the manufacturer’s protocol using the EDTA/ethanol precipitation method (ThermoFisher Scientific, Waltham, MA, USA). All sequencing reactions were resolved using capillary electrophoresis on a SeqStudio^®^ Genetic Analyzer (ThermoFisher Scientific, Waltham, MA, USA) and analyzed using DNAStar^®^-SeqMan Pro Sequence Assembly software (Lasergene^®^, version 17.4, Madison, WI, USA).

### 2.5. Statistical Analysis

Statistical analyses were performed using R statistical software (version 4.2.1, 23 June 2022, Vienna, Austria). Genotype, allele, and haplotype frequencies were computed using SHEsis online software [24]. Categorical data are presented as *N* (%) [where *N* = the number of individuals in that category and % = the frequency of individuals within that category]. Continuous data are presented as mean ± standard deviation (SD) or median ± interquartile range (IQR) [25th–75th percentile] depending on the distribution of the data. The Shapiro–Wilk test was used to assess for any deviation from normality and the Hardy–Weinberg equilibrium (HWE) was determined for each SNP for Black African and Mixed Ancestry population groups using the chi-square test with one degree of freedom. Continuous data were compared between cases and controls either using the T-test or the Mann–Whitney U-test (Wilcoxon rank-sum test) for normally and non-normally distributed data, respectively. Categorical data were compared between cases and controls using Pearson’s chi-square test or Fisher’s exact test. The main outcome of interest was RHTN. The additive model of inheritance was used to estimate associations with RHTN. Multivariable logistic regression including genetic (genotypes and haplotypes) and non-genetic variables (clinical or demographic) was used to adjust for any confounding effects. Odds ratios (ORs) described the strength of associations, and 95% confidence intervals (CI) were used as precision estimates. For all analyses, the level of statistical significance was set at *p* < 0.05.

## 3. Results

### 3.1. Demographic and Clinical Characteristics

A total of 379 participants with hypertension (29% Black African and 71% Mixed Ancestry) were successfully recruited. In 2% of the participants, adherence to antihypertensive therapy was confirmed using therapeutic drug monitoring of amlodipine levels. Table 1 compares the distribution of participant characteristics between cases and controls. The participants comprised 190 cases (with RHTN) and 189 hypertensive controls (but with no RHTN). In each group, 54% were female and the median age for cases and controls was 43.8 (20–54.2) and 42.2 (26–52.6) years, respectively (*p* = 0.05). There were statistically significant differences in the presence of comorbidities and hypertension-mediated organ damage of cases and controls. Left ventricular hypertrophy (LVH) (*p* < 0.001), chronic kidney disease (CKD) (*p* < 0.001), diabetes mellitus (*p* = 0.03), and dyslipidemia (*p* = 0.03) were more prevalent in the cases compared to controls, and aldosterone levels were significantly higher (*p* = 0.002). Use of statin or lipid-lowering and antihypertensive drugs (amlodipine, hydrochlorothiazide, atenolol, enalapril, spironolactone, and losartan) was significantly higher in cases than in controls (*p* < 0.001).

### 3.2. Genotype or Allele Frequency Distributions and Associations with Resistant Hypertension

Genotype and allele frequency distributions are shown in Table 2 and Table S2, respectively. All genotypes were in Hardy–Weinberg equilibrium (HWE) except for *ABCB1* rs2032582 and *CYP3A4* rs2740574 (*p* < 0.05) polymorphisms. We report for the first time, frequencies of *ADRB1* rs1801252 and rs1801253 polymorphisms in an African population. Allele frequencies for the SNPs under study are comparable to other African populations and variable when compared to non-African populations such as Asians and Europeans [25].

The *CYP3A5* rs776746C/C genotype (i.e., CYP3A5*3/*3) was statistically significantly more frequent in controls (29%) compared to cases (18%) (*p* = 0.03). Homozygous *CYP3A5* rs776746C/C genotype was significantly associated with reduced risk of RHTN (OR: 0.54; CI: 0.31–0.97) (Table 2). There was a trend toward a significant difference (*p* = 0.06) in the distribution of *ADRB1* rs1801252G allele carriers between cases (31%) and controls (25%). Similarly, there was a trend toward a significant difference (*p* = 0.07) in the distribution of the *NEDD4L* rs4149601A allele between cases (44%) and controls (37%). Carriers of *NEDD4L* rs4149601A allele showed a trend of more prevalence among the RHTN group (OR: 1.31; CI: 0.98–1.75) (Table S2).

### 3.3. Haplotype Frequency Distributions and Associations with Resistant Hypertension

The frequency distributions in cases or controls and associations with RHTN of haplotypes constructed from the studied SNPs, namely *ADRB1* rs1801252—rs1801253; *CYP3A5* rs776746—rs10264272—rs4130334 and *NEDD4L* rs4149601—rs292449 are shown in Table 3. Carriers of the *CYP3A5* rs776746—rs10264272—rs4130334 C–C–A haplotype were significantly (*p* = 0.03) more frequent in controls (46%) than cases (38%) and were significantly associated with reduced risk of RHTN (OR: 0.73; CI: 0.54–0.97). There was a trend towards significance (*p* = 0.06) in the distribution of the *ADRB1* rs1801252—rs1801253 G–C haplotype between cases (31%) and controls (25%) with an increased risk of RHTN (OR: 1.34; CI: 0.98–1.85). There was a trend towards significance (*p* = 0.07) in the distribution of the *NEDD4L* rs4149601—rs292449 A–C haplotype between cases (25%) and controls (19%) with an increased risk of RHTN (OR: 1.37; CI: 0.97–1.94).

### 3.4. Effect of Confounding Variables on Associations of Genetic Variables with Resistant Hypertension

Aldosterone levels, dyslipidemia, diabetes mellitus, and use of statins or lipid-lowering therapy were identified as predictor variables potentially confounding the main outcome–RHTN, at univariate analysis (*p* < 0.05) (Table 1) and prior evidence from the literature as major risk factors [26,27,28]. Associations of genotypes or haplotypes in *ADRB1*, *CYP3A5*, and *NEDD4L* polymorphisms (i.e., those with *p* < 0.05 and trended towards significance at univariate analysis) with RHTN after adjusting for aldosterone levels, dyslipidemia, diabetes mellitus and use of statins or lipid-lowering therapy are shown in Table 4. *ADRB1* rs1801252G/G genotype (*p* = 0.02; OR: 3.30; CI: 1.17–10.03), *ADRB1* rs1801252—rs1801253 G-C haplotype (*p* = 0.04; OR: 2.83; CI: 1.05–8.20) and *NEDD4L* rs4149601A/A genotype (*p* = 0.001; OR: 3.82 CI: 1.67–9.07), were significantly associated with increased risk of RHTN. The *NEDD4L* rs4149601—rs292449 A–C haplotype tended towards significant association with increased risk of RHTN (*p* = 0.08; OR: 3.14; CI: 0.88–12.9). The *CYP3A5* rs776746C/C genotype was significantly associated with reduced risk of RHTN (*p* = 0.02; OR: 0.44; CI: 0.22–0.89) while no significant association was observed for the *CYP3A5* rs776746—rs10264272—rs41303343 C–C–A haplotype (*p* = 0.59; OR: 0.86; CI: 0.49–1.50).

### 3.5. Association of Genetic Variables with Resistant Hypertension Related Adverse Outcomes: Chronic Kidney Disease (CKD) and Left Ventricular Hypertrophy (LVH)

Patients with RHTN have been shown to be at higher risk of adverse cardiovascular outcomes and target organ damage, such as CKD and LVH than patients without RHTN [29]. We hypothesized that variants associated with RHTN may be associated with adverse outcomes: CKD and LVH. Table 5 shows associations of *ADRB1* rs1801252, *CYP3A5* rs776746, and *NEDD4L* rs4149601 SNPs with CKD and LVH in unadjusted and adjusted (adjusted for the effect of aldosterone levels, dyslipidemia, diabetes mellitus and use of statins or lipid-lowering therapy) logistic regression models. The *CYP3A5* rs776746C/C genotype was significantly associated with reduced risk of CKD (unadjusted: *p* = 0.03; OR: 0.37; CI: 0.13–0.90 and adjusted: *p* = 0.04; OR: 0.29; CI: 0.08–0.93). There were no significant associations observed with LVH.

### 3.6. Assessing Blood Pressure (BP) Variation according to Genotypes in ADRB1, CYP3A5, and NEDD4L Polymorphisms in Hypertension

**Figure 1:**
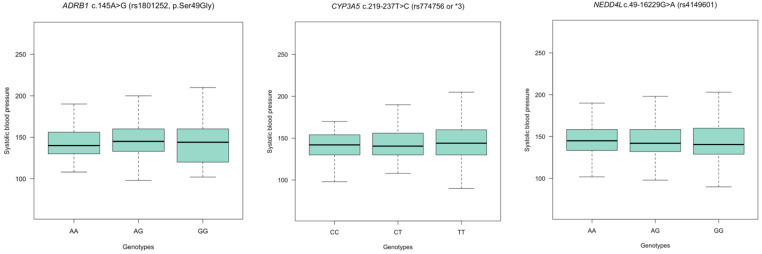
Median systolic blood pressure (SBP) variation according to genotypes. There were no significant differences in median SBP across genotypes for *ADRB1* c.145A>G (rs1801252, p.Ser49Gly), *CYP3A5* c.219-237T>C (rs774756) and *NEDD4L* c.49-16229G>A (rs4149601) polymorphisms (*ADRB1* rs1801252: A/A vs. A/G: *p* = 0.18; A/A vs. G/G: *p* = 0.91; *CYP3A5* rs774756: T/T vs. CT: *p* = 0.46; T/T vs. CC: *p* = 0.49; and *NEDD4L* rs4149601: G/G vs. A/G: *p* = 0.52; G/G vs. A/A: *p* = 0.29).

**Figure 2:**
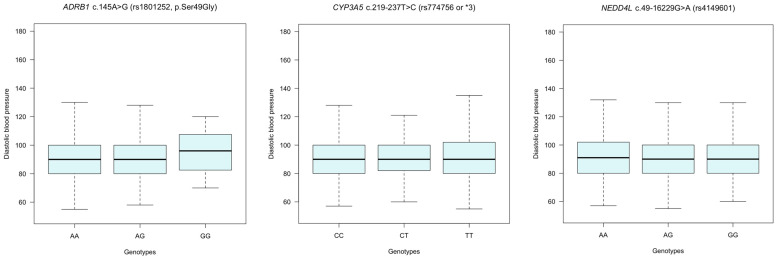
Median diastolic blood pressure (DBP) variation according to genotypes. There were no significant differences in median DBP across genotypes for *ADRB1* c.145A>G (rs1801252, p.Ser49Gly), *CYP3A5* c.219-237T>C (rs774756) and *NEDD4L* c.49-16229G>A (rs4149601) polymorphisms (*ADRB1* rs1801252: A/A vs. A/G: *p* = 0.77; A/A vs. G/G: *p* = 0.19; *CYP3A5* rs774756: T/T vs. CT: *p* = 0.55; T/T vs. CC: *p* = 0.96; and *NEDD4L* rs4149601: G/G vs. A/G: *p* = 0.73; G/G vs. A/A: *p* = 0.46).

Resistant hypertension is a phenotype defined by the number of medications and BP levels. As a secondary analysis, we also hypothesized that genetic variation in *ADRB1, CYP3A5*, and *NEDD4L* genes may explain BP variation in hypertension. Figure 1 and Figure 2 shows median systolic blood pressure (SBP) and diastolic blood pressure (DBP) variation across genotypes for *ADRB1* c.145A>G (rs1801252, p.Ser49Gly), *CYP3A5* c.219-237T>C (rs774756) and *NEDD4L* c.49-16229G>A (rs4149601) polymorphisms. Median SBP and DBP did not seem to vary significantly (*p* > 0.05) across genotype groups.

## 4. Discussion

This study set out to evaluate the role of genetic variation in *ABCB1*, *ADBR1*, *CYP3A4*, *CYP3A5*, *NEDD4L,* and *NR3C2* among South African hypertensive patients, comparing the frequency of genetic variants between RHTN and non-RHTN patients (controls). *CYP3A5* c.219-237T>C (rs774756 or CYP3A5*3) polymorphism was found to be associated with reduced risk of RHTN and protection against adverse outcomes such as CKD. *ADRB1* c.145A>G (rs1801252, p.Ser49Gly) and *NEDD4L* c.49-16229G>A (rs4149601) polymorphisms were associated with increased risk of RHTN. No significant association with RHTN was observed for polymorphisms in *ABCB1*, *CYP3A4,* and *NR3C2*.

According to Rodriguez-Antona et al. (2022), *CYP3A5* c.219-237T>C (rs774756) nucleotide change in intron 3 causes a disruption of the translational reading frame and skipping of exon 9, leading to a premature stop codon and production of a non-functional, truncated protein. At the biochemical level, the enzyme produced lacks key functional domains essential for its catalytic activity [30]. Thus, *CYP3A5* rs776746C/C genotype carriers are non-expressors while T/T genotype carriers express higher levels of CYP3A5. CYP3A5 partially metabolizes and clears CCBs such as amlodipine; some ACE inhibitors, such as enalapril; and some mineralocorticoid receptor antagonists, such as spironolactone [31]. Therefore, in non-expressors, exposure to the BP-lowering effect of CYP3A5 substrate drugs is increased as there is reduced metabolic clearance. As such, it is possible that *CYP3A5* rs776746C allele carriers may derive greater benefit from antihypertensive drugs that are CYP3A5 substrates, which also results in reduced occurrence of hypertension-related adverse outcomes such as CKD. Furthermore, carriage of the *CYP3A5* rs776746C allele has been shown to be associated with reduced clearance and increased exposure to drugs metabolized by CYP3A5 in several pharmacokinetic studies [13] further supporting findings from this study.

CYP3A4 and CYP3A5 have broad overlapping substrate specificities and according to Soria-Chacartegui et al. (2023), CYP3A4 contributes mainly to the metabolic clearance of amlodipine [32]. However, we observed no associations between the studied *CYP3A4* polymorphism and RHTN. To our knowledge, there have not been reports of the association of *CYP3A4* polymorphisms with RHTN, although *CYP3A4* rs2740574 polymorphism has been shown to be associated with BP response to amlodipine in African Americans [33].

The *ADRB1* rs1801252G/G genotype and *ADRB1* rs1801252–1801253 G–C haplotype were associated with an increased likelihood of RHTN. Beta-blockers, such as atenolol, metoprolol, or bisoprolol, result in a lowering of BP through inhibition of the downstream effects of endogenous catecholamines such as epinephrine and norepinephrine. Beta-blockers achieve this by blocking beta-adrenergic receptors, which in turn reduces heart rate, cardiac output, and the release of renin from the kidneys [34]. However, polymorphisms such as *ADRB1* rs1801252 and *ADRB1* rs1801253 can stunt this effect. For example, the *ADRB1* rs1801252G allele has been reported to increase agonist-promoted downregulation due to altered N-glycosylation, affecting receptor stability and function. Conversely, the *ADRB1* rs1801253C allele alters G-protein coupling, leading to decreased adenylyl cyclase activity. This results in a reduced conversion of ATP to cAMP, a critical messenger in the signal transduction pathway that mediates the effects of beta-adrenergic stimulation. Consequently, the generation of cAMP from ATP is diminished, leading to reduced physiological responses to beta-blockers [35]. While there have been inconsistent findings across populations on associations of the *ADRB1* polymorphisms and response to beta-blockers, previous studies indicate that *ADRB1* rs1801252A allele carriers respond better to beta-blocker treatment compared to G allele carriers, and individuals carrying the G–C haplotype (*ADRB1* rs1801252–1801253) exhibit a poor response [35]. This was consistent with our study’s outcomes, so it is possible that observed associations with RHTN are due to poor response to beta-blockers.

The carriers of the *NEDD4L* rs4149601A/A genotype were observed to have an increased risk of RHTN. Under normal physiological functions, NEDD4L serves as a principal regulator of sodium ion transporters in the kidney, which are targeted by antihypertensive medications such as hydrochlorothiazide (HCTZ) and amiloride. For example, NEDD4L is a specific ubiquitin ligase of the epithelial sodium channel (ENaC). NEDD4L has been reported to be composed of a C2-WW(x4)-HECT domain which facilitates binding to the PY motif of the ENaC leading to channel ubiquitination and endocytosis. This in turn suppresses ENaC function and reduces sodium reabsorption in the kidney, which lowers blood volume and blood pressure [16]. The presence of the *NEDD4L* rs4149601A allele causes alternative splicing, resulting in a non-functional NEDD4L [18]. The *NEDD4L* rs4149601A allele has been linked to adverse cardiovascular outcomes due to poor response in patients on HCTZ in several studies. For example, according to McDonough et al., (2016), carriers of the *NEDD4L* rs4149601A allele were found to not respond well to treatment with HCTZ, had less BP reduction, and were at increased risk of adverse CVD outcomes compared to G allele carriers. Therefore, these prior associations with poor response to HCTZ resulting in adverse outcomes due to *NEDD4L* rs4149601, are also consistent with findings from this study, although this study establishes a link with RHTN.

The genes selected for analysis in this study encode key enzymes involved in the pharmacokinetics and pharmacodynamics of antihypertensive drugs. We observed that selected polymorphisms in *ABCB1*, *CYP3A4,* and *NR3C2* genes were not significantly associated with RHTN, despite prior evidence of their association with antihypertensive drug response in other populations [9]. We speculate that this lack of significance could be due to genetic heterogeneity that exists between population groups, meaning that the genetic makeup affecting drug response might vary significantly among different ethnicities. It is possible that the specific variants selected for this study may not be the most relevant ones for South Africans (i.e., Black Africans and Mixed Ancestry in this instance).

Our study has limitations. First, the accuracy of our findings was dependent on being able to rule out non-adherence to antihypertensive therapy. The adherence was self-reported, which is the least reliable method. Where applicable, amlodipine concentrations were measured when non-adherence was suspected. Second, all participants recruited for the study had hypertension including the controls. The absence of a normotensive control group could potentially affect the interpretation and generalizability of the study findings, as there is no reference point for comparison to assess the impact of hypertension on the variables under investigation. Third, we did not adjust for multiple testing. Fourth, RHTN is a polygenic disorder, and this study was a targeted and hypothesis-driven approach, focusing on a few selected genes or variants. It is possible that our approach could have fallen short in detecting other variants in genes with unknown biological links to hypertension, necessitating the need for extensive research.

In conclusion, RHTN is a phenotype resulting from poor antihypertensive drug response. It appears that *ADRB1* c.145A>G (rs1801252, p.Ser49Gly), *CYP3A5* c.219-237T>C (rs774756 or CYP3A5*3) and *NEDD4L* c.49-16229G>A (rs4149601) polymorphisms may be of pharmacogenomic importance in African populations. Our findings suggest that screening for these variants may help in predicting patients who are likely to have good or poor responses to antihypertensive drugs among Africans. For example, curating these variants in the development of rapid diagnostic tests (i.e., bedside tests) and dosing algorithms, may assist clinicians in making rapid decisions about the selection and dosage of antihypertensive therapy, thereby improving daily medical practice. In addition to aiding in diagnosis, variants may potentially aid in the development of efficacious alternative antihypertensive drugs other than the ones affected by these polymorphisms. However, further research is still needed. It is important that our findings be replicated in larger prospective cohort or pharmacokinetic studies to establish causality. Additionally, research in other African populations is necessary, as our study was limited to Black African and Mixed Ancestry individuals from South Africa. Furthermore, non-targeted approaches such as genome-wide association studies (GWAS) or whole-exome sequencing may aid in the possible discovery of African-specific variants associated with refractory response to antihypertensive therapy.

## Figures and Tables

**Table 1:** Demographic and clinical characteristics of study participants.

Variable(s)	Cases (N = 190)	Controls (N = 189)	*p*-Value
Age years	43.8 (20–54.2)	42.2 (26–52.6)	0.05
Female sex	103 (54.2%)	102 (54.0%)	0.99
Ethnicity			
Black African	58 (30.5%)	52 (27.5%)	0.57
Mixed ancestry	132 (69.5%)	137 (72.5%)	
Alcohol consumption (frequent/occasional)	45 (23.7%)	47 (24.9%)	0.90
Smoking (active/past)	63 (33.2%)	64 (33.9%)	0.99
Positive family History of hypertension	123 (64.7%)	121 (64.0%)	0.91
Blood pressure (mmHg)			
Uncontrolled (≥140/90)	149 (78.4%)	68 (36.0%)	<0.001
Controlled (<140/90)	41 (21.6%)	121 (64.0%)	
Left ventricular hypertrophy ^a^			
Yes	90 (47.4%)	52 (27.5%)	<0.001
No/unknown	100 (52.6%)	189 (72.5%)	
Aldosterone (pmol/L)	276 (31–435)	200 (2.3–313)	0.002
Comorbidities			
Chronic kidney disease (CKD) ^b^	36 (19.1%)	10 (5.3%)	<0.001
Diabetes mellitus (DM)	33 (17.6%)	18 (9.6%)	0.03
Dyslipidemia	30 (15.8%)	15 (7.9%)	0.03
Ischemic heart disease (IHD)	17 (8.9%)	9 (4.8%)	0.11
Previous stroke	13 (6.8%)	5 (2.6%)	0.09
Concomitant drugs			
Statin or lipid-lowering therapy	76 (40.0%)	45 (23.8%)	<0.001
Diabetic treatment ^c^	23 (12.1%)	15 (7.9%)	0.08
Analgesics	12 (6.0%)	10 (5.2%)	0.69
Antihypertensives drugs			
Amlodipine	172 (90.5%)	116 (61.3%)	<0.001
Hydrochlorothiazide	148 (77.9%)	108 (57.1%)	<0.001
Atenolol	123 (64.7%)	27 (14.2%)	<0.001
Enalapril	150 (78.9%)	88 (46.6%)	<0.001
Spironolactone	52 (27.3%)	7 (3.7%)	<0.001
Losartan	39 (20.5%)	14 (7.4%)	<0.001

^a^ Abnormal LVH: females > 95 g/m^2^, males > 115 g/m^2^; ^b^ CKD: eGFR MDRD < 60 mL/min; ^c^ Diabetic treatment included metformin, insulin, or gliclazide.

**Table 2:** Genotype frequency distributions between cases and controls.

Single Nucleotide Polymorphism	Genotype	Genotype Frequencies, *N* (freq)			*p*-Value	OR [95% CI]
		Combined	Cases	Controls		
*ABCB1* rs1045642 (c.3435C>T)	C/C	191 (0.50)	99 (0.52)	92 (0.49)	1	
	C/T	142 (0.37)	70 (0.37)	72 (0.38)	0.66	0.90 [0.57–1.42]
	T/T	46 (0.12)	21 (0.11)	25 (0.13)	0.51	0.78 [0.39–1.57]
*ABCB1* rs2032582 (c.2677C>A)	C/C	219 (0.59)	112 (0.60)	107 (0.58)	1	
	A/C	109 (0.29)	54 (0.29)	55 (0.30)	0.82	0.93 [0.58–1.52]
	A/A	46 (0.13)	22 (0.11)	24 (0.13)	0.74	0.88 [0.44–1.74]
*ADRB1* rs1801252 (c.145A>G)	A/A	190 (0.51)	92 (0.48)	107 (0.57)	1	
	A/G	149 (0.40)	79 (0.42)	70 (0.37)	0.23	1.31 [0.84–2.05]
	G/G	31 (0.08)	19 (0.10)	12 (0.06)	0.13	1.83 [0.80–4.39]
*ADRB1* rs1801253 (c.1165G>C)	C/C	195 (0.51)	100 (0.53)	95 (0.50)	1	
	C/G	153 (0.40)	74 (0.39)	79 (0.42)	0.67	0.89 [0.57–1.39]
	G/G	31 (0.08)	16 (0.08)	15 (0.08)	0.98	1.01 [0.44–2.34]
*CYP3A4* rs2740574 (c.-392C>T, CYP3A4*1B)	T/T	136 (0.36)	59 (0.32)	77 (0.41)	1	
	C/T	132 (0.35)	74 (0.40)	58 (0.31)	0.05	1.30 [0.78–2.17]
	C/C	105 (0.28)	52 (0.28)	53 (0.28)	0.36	0.78 [0.47–1.30]
*CYP3A5* rs776746 (c.219-237T>C, CYP3A5*3)	T/T	144 (0.38)	78 (0.41)	66 (0.35)	1	
	C/T	146 (0.39)	77 (0.41)	69 (0.37)	0.82	0.94 [0.58–1.54]
	C/C	89 (0.23)	35 (0.18)	54 (0.29)	0.03	0.54 [0.31–0.97]
*CYP3A5* rs10264272 (*c.624C>T*, CYP3A5*6)	C/C	285 (0.75)	141 (0.74)	144 (0.76)	1	
	C/T	86 (0.22)	45 (0.24)	41 (0.22)	0.71	1.12 [0.67–1.87]
	T/T	8 (0.10)	4 (0.02)	4 (0.02)	0.99	1.02 [0.18–5.60]
*CYP3A5* rs41303343 (insT, CYP3A5*7)	A/A	322 (0.85)	158 (0.83)	164 (0.87)	1	
	A/T	52 (0.14)	30 (0.16)	22 (0.12)	0.29	1.41 [0.75–2.69]
	T/T	3 (0.01)	1 (0.01)	2 (0.01)	0.99	0.51 [0.01–10.08]
*NEDD4L* rs4149601 (c.49-16229G>A)	G/G	134 (0.36)	59 (0.31)	75 (0.40)	1	
	A/G	179 (0.47)	95 (0.50)	84 (0.45)	0.14	1.43 [0.89–2.31]
	A/A	64 (0.17)	36 (0.19)	28 (0.15)	0.13	1.63 [0.86–3.11]
*NEDD4L* rs292449 (c.-300G>C)	G/G	100 (0.26)	52 (0.28)	48 (0.25)	1	
	C/G	190 (0.50)	89 (0.47)	101 (0.53)	0.25	0.73 [0.42–1.26]
	C/C	88 (0.23)	48 (0.25)	40 (0.21)	0.77	0.90 [0.49–1.67]
*NR3C2* rs5522 (c.538G>A)	T/T	286 (0.76)	145 (0.76)	141 (0.75)	1	
	C/T	90 (0.24)	45 (0.24)	45 (0.24)	0.99	0.97 [0.59–1.61]
	C/C	1 (0.003)	0 (0)	1 (0.01)	0.49	0 [0–38.2]
*NR3C2* rs2070950 (c.-2-358C>G)	G/G	171 (0.45)	88 (0.47)	83 (0.44)	1	
	C/G	145 (0.38)	71 (0.38)	74 (0.39)	0.73	0.91 [0.57–1.44]
	C/C	62 (0.16)	30 (0.16)	32 (0.17)	0.77	0.88 [0.47–1.65]

**Table 3:** Haplotype frequency distributions between cases and controls.

SNP Combination	Haplotype	Cases, *N* (Freq)	Controls, *N* (Freq)	*p*-Value	OR [95% CI]
*ADRB1* rs1801252—rs1801253	A-C	157 (0.41)	175 (0.46)	0.17	0.82 [0.61–1.09]
	A-G	106 (0.28)	109 (0.29)	0.77	0.96 [0.69–1.31]
	G-C	117 (0.31)	94 (0.25)	0.06	1.34 [0.98–1.85]
*CYP3A5* rs776746—rs10264272—rs41303343	C-C-A	144 (0.38)	172 (0.46)	0.03	0.73 [0.54–0.97]
	T-C-A	150 (0.40)	130 (0.35)	0.14	1.25 [0.93–1.68]
	T-C-T	30 (0.08)	25 (0.07)	0.43	1.25 [0.72–2.16]
	T-T-A	50 (0.13)	46 (0.12)	0.65	1.10 [0.72–1.70]
*NEDD4L* rs4149601—rs292449	A-C	93 (0.25)	72 (0.19)	0.07	1.37 [0.97–1.94]
	A-G	73 (0.19)	68 (0.18)	0.70	1.07 [0.75–1.55]
	G-C	100 (0.26)	123 (0.33)	0.05	0.73 [0.54–1.00]
	G-G	112 (0.30)	111 (0.30)	0.99	0.99 [0.73–1.37]

**Table 4:** Multivariable logistic regression analysis adjusting for confounding variables.

SNP/SNP Combination	Genotype/Haplotype	Coefficient	*p*-Value	OR [95% CI] ^a^
*ADRB1* rs1801252	GG	1.19	0.02	3.30 [1.17–10.03] ^b^
*ADRB1* rs1801252—rs1801253	G-C	1.04	0.04	2.83 [1.05–8.20] ^b^
*CYP3A5* rs776746	CC	−0.80	0.02	0.44 [0.22–0.89] ^b^
*CYP3A5* rs776746—rs1026427—rs41303343	C-C-A	−0.14	0.59	0.86 [0.49–1.50]
*NEDD4L* rs4149601	AA	1.34	0.001	3.82 [1.67–9.07] ^b^
*NEDD4L* rs4149601—rs292449	A-C	1.14	0.08	3.14 [0.88–12.9]

^a^ ORs for genotypes and haplotypes were adjusted for aldosterone levels, dyslipidemia, diabetes mellitus, and use of statins or lipid-lowering therapy, ^b^ statistically significant associations with resistant hypertension.

**Table 5:** Association with chronic kidney disease (CKD) and left ventricular hypertrophy (LVH).

		Chronic Kidney Disease					
		Unadjusted			Adjusted ^a^		
Variable	Genotype	Coefficient	*p*-Value	OR [95% CI]	Coefficient	*p*-Value	OR [95% CI]
*ADRB1* *rs1801252*	G/G	−0.11	0.87	0.90 [0.20–2.83]	−0.09	0.83	0.91 [0.39–2.08]
*CYP3A5* *rs776746*	C/C	−0.99	0.03	0.37 [0.13–0.90]	−1.22	0.04	0.29 [0.08–0.93] ^b^
*NEDD4L* *rs4149601*	A/A	0.19	0.71	1.20 [0.46–3.54]	−0.25	0.72	0.78 [0.16–3.02]
		**Left Ventricular Hypertrophy**					
*ADRB1* *rs1801252*	G/G	0.26	0.12	1.29 [0.83–2.00]	0.55	0.25	1.13 [0.57–2.25]
*CYP3A5* *rs776746*	C/C	−0.08	0.76	0.92 [0.53–1.59]	0.12	0.72	1.74 [0.67–4.45]
*NEDD4L* *rs4149601*	A/A	−0.05	0.85	0.95 [0.50–1.74]	−0.17	0.69	0.85 [0.37–1.89]

^a^ ORs for genotypes were adjusted for aldosterone levels, dyslipidemia, diabetes mellitus, and use of statins or lipid-lowering therapy, ^b^ statistically significant associations with CKD.

## Data Availability

The data that support the findings of this study are available from the corresponding author [C.D] upon reasonable request.

## Supplementary Materials

The following supporting information can be downloaded at: https://www.mdpi.com/article/10.3390/jpm14070664/s1, Table S1: Primer sequences and annealing temperatures; Table S2: Allele frequency distributions between cases and controls. References [36,37,38,39,40] are cited in the Supplementary Materials.
