# Supplementary material for: Genetic Variation in ABCB1, ADRB1, CYP3A4, CYP3A5, NEDD4L and NR3C2 Confers Differential Susceptibility to Resistant Hypertension among South Africans
Source: J Pers Med. 2024 Jun 21;14(7):664. doi: 10.3390/jpm14070664 (PMC11277774; doi:10.3390/jpm14070664)
Supplement: Supplementary file 1 [file jpm-14-00664-s001.zip › jpm-3046435-supplementary.pdf]

Journal of Personalized Medicine

Table S1: Primer sequences and annealing temperatures

SNP	Forward Primer (5′ – 3′)	Reverse Primer (5′ – 3′)	Ta (°C)	PCR Product (bp)	Ref
ABCB1 rs1045642 (c.3435C>T)	TGCTGGTCCTGAAGTTGATCTGTGAAC	ACATTAGGCAGTGACTCGATGAAGGCA	60	248	[36]
ABCB1 rs2032582 (c.2677C>A)	ATGGTTGGCAACTAACACTGTTA	AGCAGTAGGGAGTAACAAAATAACA	54	206	[37]
ADRB1 rs1801252 (c.145A>G)	GACCTCCCTCTGCGCACCAC	CTGAGGTCCACAGCTCGCAGA	61	508	[38]
ADRB1 rs1801253 (c.1165G>C)	ACGCTGGGCATCATCATGGGC	CTGAGGTCCACAGCTCGCAGA	57	332	[38]
CYP3A4 rs2740574 (c.-392C>T)	GGACAGCCATAGARACAAGGGCT	AGGTTTCCATGGCCAAGTCT	64	334	[39]
CYP3A5 rs776746 (c.219 237A>G)	CATCAGTTAGTAGACAGATGA	GGTCCAAACAGGGAAGAAATA	51	293	[40]
CYP3A5 rs10264272 (c.624G>A)	TGGAAGATGATTCAGCAGATA	GTGGGGTGTTGACAGCTAAAG	58	495	[40]
CYP3A5 rs41303343 (27131_27132insT)	CTTCAATAGTACTGCATGGAC	CTGTACCACGGCATCATAGCT	53	108	[40]
NEDD4L rs4149601 (c.49-16229G>A)	CGACTTCCGCATACTCTTCAG	CTGTCACGGTGTTCCTACATT	60	417	designed
NEDD4L rs292449 (c.-300G>C)	CCTCTTGTTCAAACTCCCTAAGA	TCTGTCCATCGTGAAGCATAC	60	239	designed
NR3C2 rs5522 (c.538C>T)	TAACGGACTTGAGAGAGGAGAG	CCTATGAGCAGCAGAACCAA	60	443	designed
NR3C2 rs2070950 (c.-2-358C>G)	CTTTGGTCTCCATCGCTAACA	CAAGCCACCCACTTCACTAA	60	398	designed

Journal of
Personalized Medicine

MDPI

Table S2: Allele frequency distributions between cases and controls

SNP	Variant Allele	Allele Frequencies, *N* (freq)			P-Value	OR [95% CI]
		Combined	Cases	Controls		
ABCB1 rs1045642 (c.3435C>T)	T	234 (0.31)	112 (0.30)	122 (0.32)	0.40	1.14 [0.83 – 1.55]
ABCB1 rs2032582 (c.2677C>A)	A	201 (0.27)	98 (0.26)	103 (0.28)	0.61	0.92 [0.67 – 1.27]
ADRB1 rs1801252 (c.145A>G)	G	216 (0.28)	117 (0.31)	99 (0.25)	0.06	1.46 [0.97 – 2.19]
ADRB1 rs1801253 (c.1165G>C)	G	215 (0.29)	106 (0.28)	109 (0.29)	0.77	1.04 [0.76 – 1.44]
CYP3A4 rs2740574 (c.-392C>T, *CYP3A4*1B)*	C	342 (0.46)	178 (0.48)	164 (0.44)	0.21	1.19 [0.89 – 1.59]
CYP3A5 rs776746 (c.219-237T>C; *CYP3A5*3)*	C	326 (0.43)	142 (0.37)	184 (0.49)	0.02	0.62 [0.47 – 0.84]
CYP3A5 rs10264272 (c.624C>T, *CYP3A5*6)*	T	102 (0.14)	48 (0.13)	54 (0.14)	0.50	1.15 [0.76 – 1.75]
CYP3A5 rs41303343 (insT, *CYP3A5*7)*	T	57 (0.08)	32 (0.08)	25 (0.07)	0.37	0.78 [0.45 – 1.34]
NEDD4L rs4149601 (c.49-16229G>A)	A	307 (0.39)	167 (0.44)	140 (0.37)	0.07	1.31 [0.98 – 1.75]
NEDD4L rs292449 (c.-300G>C)	G	366 (0.49)	185 (0.48)	181 (0.49)	0.77	0.95 [0.72 – 1.28]
NR3C2 rs5522 (c.538C>T)	C	92 (0.13)	45 (0.12)	47 (0.13)	0.76	0.93 [0.60 – 1.45]
NR3C2 rs2070950 (c.-2-358C>G)	C	269 (0.36)	131 (0.35)	138 (0.37)	0.48	0.92 [0.68 – 1.24]
